# A First Principles Study of H_2_ Adsorption on LaNiO_3_(001) Surfaces

**DOI:** 10.3390/ma10010036

**Published:** 2017-01-05

**Authors:** Changchang Pan, Yuhong Chen, Na Wu, Meiling Zhang, Lihua Yuan, Cairong Zhang

**Affiliations:** 1 State Key Laboratory of Advanced Processing and Recycling of Non-ferrous Metals, Lanzhou University of Technology, Lanzhou 730050, China; panchangchang@fjirsm.ac.cn (C.P.); 18794217051@163.com (N.W.); zhcrxy@lut.cn (C.Z.); 2 School of Science, Lanzhou University of Technology, Lanzhou 730050, China; zhangml_2000@126.com (M.Z.); yuanlh@lut.cn (L.Y.); 3 The School of Nuclear Science and Technology, Lanzhou University, Lanzhou 730000, China

**Keywords:** density functional theory, LaNiO_3_(001), surface adsorption, conductivity

## Abstract

The adsorption of H_2_ on LaNiO_3_ was investigated using density functional theory (DFT) calculations. The adsorption sites, adsorption energy, and electronic structure of LaNiO_3_(001)/H_2_ systems were calculated and indicated through the calculated surface energy that the (001) surface was the most stable surface. By looking at optimized structure, adsorption energy and dissociation energy, we found that there were three types of adsorption on the surface. First, H_2_ molecules completely dissociate and then tend to bind with the O atoms, forming two –OH bonds. Second, H_2_ molecules partially dissociate with the H atoms bonding to the same O atom to form one H_2_O molecule. These two types are chemical adsorption modes; however, the physical adsorption of H_2_ molecules can also occur. When analyzing the electron structure of the H_2_O molecule formed by the partial dissociation of the H_2_ molecule and the surface O atom, we found that the interaction between H_2_O and the (001) surface was weaker, thus, H_2_O was easier to separate from the surface to create an O vacancy. On the (001) surface, a supercell was constructed to accurately study the most stable adsorption site. The results from analyses of the charge population; electron localization function; and density of the states indicated that the dissociated H and O atoms form a typical covalent bond and that the interaction between the H_2_ molecule and surface is mainly due to the overlap-hybridization among the H 1s, O 2s, and O 2p states. Therefore, the conductivity of LaNiO_3_(001)/H_2_ is stronger after adsorption and furthermore, the conductivity of the LaNiO_3_ surface is better than that of the LaFeO_3_ surface.

## 1. Introduction

ABO_3_ perovskites are a group of inexpensive materials that possess high capacities; fast charge and discharge capabilities; and universally present the phenomenon of hydrogen storage. Therefore, perovskites have been systematically investigated as cathodes for nickel/metal hydride (Ni/MH) batteries. Thus, these materials have an important potential application value [1,2]. In recent years, many studies have been devoted to the investigation of the chemical properties of ABO_3_ perovskites, both experimentally and theoretically. Deng et al. [1] prepared LaFeO_3_ using a stearic acid combustion method and investigated the structure, chemical properties and hydrogen storage mechanism of LaFeO_3_ using X-ray diffraction (XRD), X-ray photoelectron spectroscopy (XPS) and mass spectrometry (MS), coupled with pressure composition temperature (PCT) methods; the analysis of the results showed that the discharge capacity was 626 mAh/g at 80 °C; however, Wang et al. [3] calculated the discharge capacity of LaFeO_3_ to be 662.4 mAh/g at 60 °C using a first principles method, where the maximum value of the discharge capacity increased with increasing temperature. Wærnhus et al. [4] reported on the electrical conductivity of polycrystalline LaFeO_3_ as a function of the thermal properties of the materials; and that the conductivity of LaFeO_3_ was affected by annealing for extended periods at temperatures above 1000 °C, prior to the conductivity measurements. Although the discharge capacity of LaFeO_3_ was sufficiently large enough for its use as a cathode in Ni/MH batteries, the required temperature was too high and had poor conductivity [1,3]. Hansmann et al. [5,6,7,8] reported that LaNiO_3_ materials possessed good conductivity. Kleperis et al. [9] focused on the discharge capacity of LaNiO_3_, which was also 360 mAh/g, according to theoretical works. Hsiao and Qi [10] reported that thin films of LaNiO_3–*x*_ had good electrical conductivity when the sintering temperature was 600 °C; and the epitaxial films, particularly under tensile strain, presented higher stability. Although the discharge capacity of LaNiO_3_ is less than that of LaFeO_3_, LaNiO_3_ has better conductivity [11,12]. Kohn et al. [13] investigated the electronic structure of LaNiO_3_ using first principles density functional theory (DFT) calculations. Guan et al. [14,15] investigated the electronic structure of LaNiO_3_ using first principles calculations, and then calculated the surface energy of LaNiO_3_(001) and studied the electronic structure of the surface. Due to its good conductivity; high chemical stability; and high catalytic activity, LaNiO_3_ is often used in the manufacture of thin film electrode materials, electron emitters, and catalysts [11,16,17,18].

To correctly understand the changes in the microstructure of hydrogen storage materials and recognize the hydrogen storage properties of LaNiO_3_, it is important to research the hydrogen storage process for LaNiO_3_. The DFT evaluation is based on the plane wave expansion on this work. Starting from the surface of LaNiO_3_, the most stable surface was determined by calculating the surface energy. Then, the adsorption properties of H_2_ on the surface were investigated and included looking at changes in the electronic structure; electron and bond populations; and the change in electrical conductivity before and after adsorption, for the hydrogen storage process. The results were then compared with the existing experimental and theoretical data, and this comparison provides the corresponding microscopic mechanism and theoretical basis for further studies.

## 2. Models and Computational Methods

### 2.1. Calculation Parameters and Models

**Figure 1:**
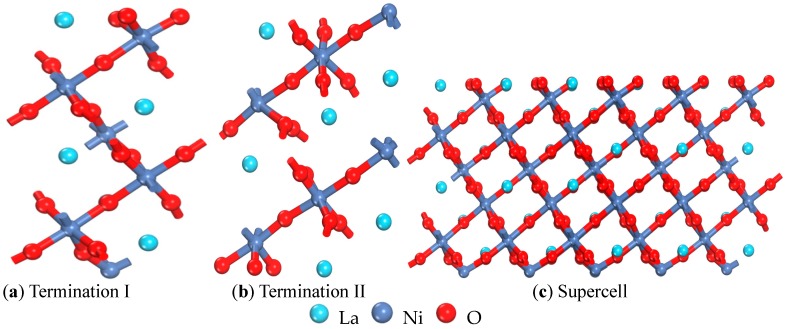
The models of the LaNiO_3_(001) surface. (**a**) The LaO-terminated LaNiO_3_(001) is Termination I; (**b**) The Ni-terminated LaNiO_3_(001) is Termination II; (**c**) The supercell of Termination I.

The first principles calculations were performed using the Cambridge Sequential Total Energy Package (CASTEP 7.1) computer code [19] in the framework of DFT, and the DFT evaluation was based on the plane-wave expansion. The generalized gradient approximation (GGA) [20] in the form of the Perdew-Burke-Ernzerhof function for exchange-correlation potential and the ultrasoft pseudopotential [21] are described for the electron-ion interaction. We treated the O (2s, 2p), Ni (3s, 3p, 3d, 4s), and La (5s, 5p, 5d, 6s) electrons as valence states, whereas the remaining electrons were kept frozen as core states. The partial occupancies were calculated using Finite temperature approaches—smearing methods—and the smearing was 0.1 eV. As the LaNiO_3_ crystal is a rhombohedral perovskite (R-3c), two models were used to study the properties of LaNiO_3_: LaO-terminated (Figure 1a, termination I) and Ni-terminated (Figure 1b, termination II). Due to calculation accuracy and computational efficiency considerations, the necessary convergence test for the cutoff energy and k-point mesh was performed, and all calculations were conducted using a cutoff energy of 600 eV and a 9 × 9 × 1 k-point mesh in the Brillouin zone, which is used for a 1 × 1 unit cell of the perovskite formula unit containing a total of 40 atoms. The results of the convergence test indicated that the model can meet the computational conditions. Considering computational accuracy, the 3 × 3 supercell was adopted (Figure 1c) to investigate the electronic structure of the most stable adsorption position. The k-point density was maintained as close to this value as possible for different slab calculations, and the convergence criteria for energy and displacement were 2.0 × 10^−5^ eV/atom and 10^−3^ Å, respectively. The vacuum region was 10 Å thick to ensure that the vacuum thickness was large enough to avoid spurious interactions between the slabs as well as to verify that the electrostatic potential was flat in the vacuum region for each result. The optimum lattice parameters (*a* = *b* = 5.3908 Å, *c* = 13.1074 Å) for LaNiO_3_ deviate slightly from the experimental values (*a* = *b* = 5.4534 Å, *c* = 13.1369 Å) [22], and indicates that the model can guarantee accurate calculations.

### 2.2. Calculations of Surface Energy

Surface energy can provide important information regarding the structural stability of the surface. According to the definition of Chiou Jr. and Carter [23], the surface energy density of a solid, which corresponds to the energy variation (per unit area) due to the creation of a surface, is given by

*E*_surf_ = (*E*_slab_ − *NE*_bulk_)/2*A* (1)

where *E*_slab_ and *E*_bulk_ represent the total energy of the slab and the bulk total energy per LaNiO_3_ unit, respectively. *N* and *A* indicate the number of LaNiO_3_ units in the slab and the surface area of the slab, respectively.

The calculated surface energies of different LaNiO_3_ surfaces are presented in Table 1. The results indicate that the (001) surface possesses the lowest surface energy; therefore, the (001) surface is considered to be the most stable surface, which is similar to that in Reference [15] where the surface energy for the LaO-terminated (001) surface is approximately 2.03 eV/Å^2^. Choi et al. [24] and Evarestov et al. [25] also mention that the LaNiO_3_(001) surface is generally the most stable surface in perovskites. Thus, the LaNiO_3_(001) surface was investigated for hydrogen storage.

### 2.3. Calculations of Adsorption Energy and Dissociation Energy on the LaNiO_3_(001) Surface

Based on the analysis of the adsorption and dissociation energies on the (001) surface, the most stable adsorption site and related properties were investigated. The adsorption energy was defined with the following equation [26]:

(2) $$E_{\mathrm{ads}}=E_{\mathrm{slab}/H_{2}}-E_{\mathrm{clean}}-E_{H_{2}}$$

where $E_{\mathrm{clean}}$ and $E_{\mathrm{slab}/H_{2}}\;$are the total energies of LaNiO_3_(001) and LaNiO_3_(001)/H_2_, respectively. $E_{H_{2}}\;$is the total energy of a H_2_ molecule. In terms of this definition, a negative value corresponds to an exothermic process and indicates a stable structure. Moreover, $E_{\mathrm{dis}}\;$is the dissociation energy and can be expressed as the following equation:

(3) $$E_{\mathrm{dis}}=2E_{H}-E_{H_{2}}$$

where $E_{H}$ is the energy of a H atom. The dissociation energy of H_2_ is smaller than that of free H_2_, which indicates that H_2_ presents a dissociation phenomenon. A negative value shows that the H_2_ molecule has been completely dissociated and the smaller value indicates that dissociation is more abundant for H_2_.

## 3. Results and Discussion

### 3.1. Analysis of Surface Adsorption Sites

**Figure 2:**
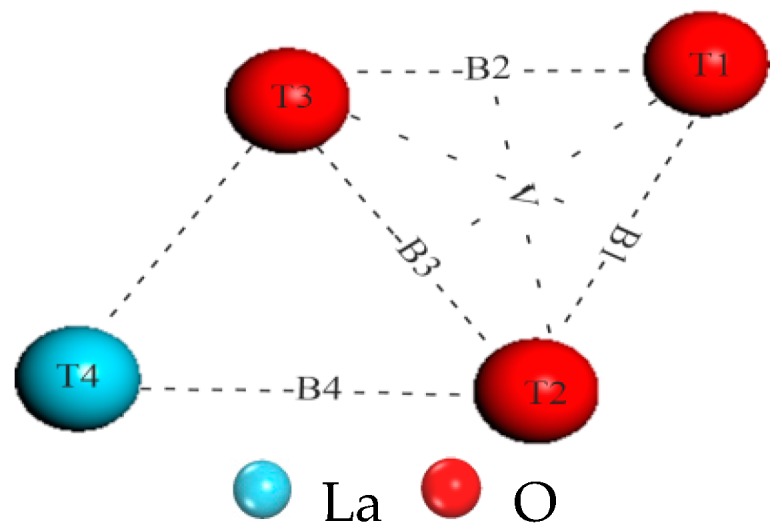
The initial adsorption positions for the LaNiO_3_(001)/H_2_ system in Termination I.

**Figure 3:**
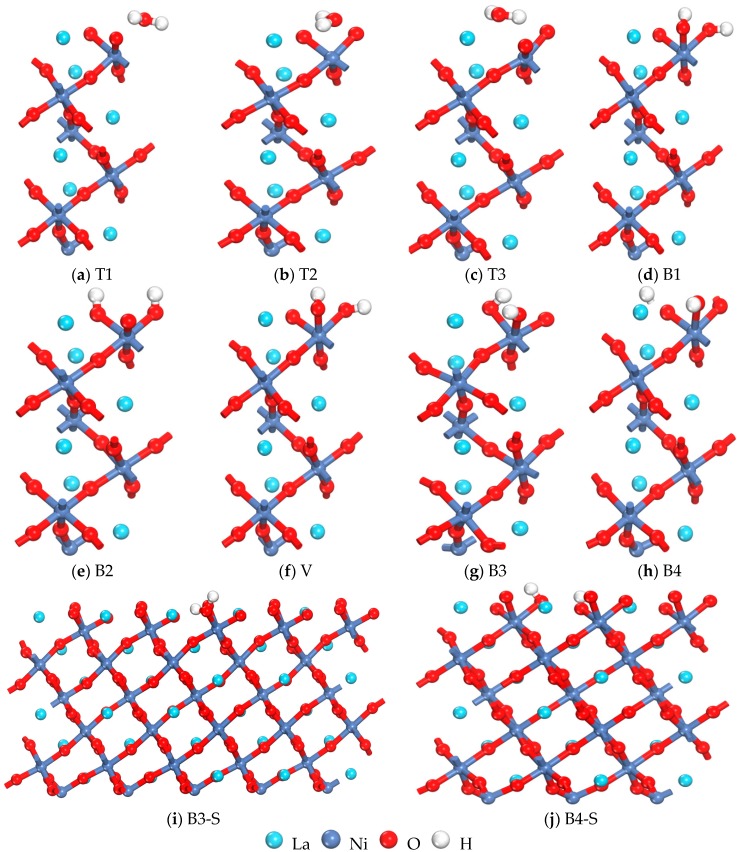
The optimized geometrical structure of LaNiO_3_(001)/H_2_ in Termination I.

All the possible adsorption sites for H_2_ in Termination I are shown in Figure 2. T1, T2 and T3 represent the top of O; T4 corresponds to the top of La; B1, B2 and B3 indicate the O; B4 corresponds to the La bridge; and V is a hollow site. T5 corresponds to the top of Ni in Termination II. As shown in Table 2 and Figure 3, the calculated adsorption energy *E*_ads_ and dissociation energy *E*_dis_ of different positions for the (001) surface are listed based on the previous definitions, and the minimum distance between H atoms and surface atoms after adsorption are also included (*r*_H–H_, *r*_H–O_, *r*_H–La_, and *r*_H–Ni_). The calculated *r*_H–H_ and *E*_dis_ for free H_2_ were 0.752 Å and −4.54 eV, respectively, and the result mostly agreed with the experimental values (0.752 Å and −4.48 eV) [27]. The calculated results indicated that when the H_2_ molecule was located on the B1, B2, B3 and V sites in Termination I, the calculated *r*_H–H_ was clearly large and the *E*_dis_ presented a negative value after geometry optimization, which showed that the H_2_ molecule had been dissociated and that the two H atoms approached the top of O and formed two –OH– with O atoms (as shown in Figure 3). The calculated *E*_ads_ is significantly larger than −40 kJ/mol^−1^ (*E*_ads_ is −0.415 eV for a H_2_ molecule), which indicates that this adsorption is a strong chemical adsorption [28] on these sites. The *E*_ads_ is the largest on B3, which means that the LaNiO_3_(001)/H_2_ system achieved the most stable structure on B3. For computational accuracy, the 3 × 3 supercell was adopted (Figure 3i); moreover, the H_2_ molecule was located on the T1, T2 and T3 sites where two optimized H atoms approached an O atom to form a H_2_O molecule (Figure 3a–c). These structures were similar to the value that Lie and Clementi [29] used to calculate the geometric parameters of a H_2_O molecule (*r*_H–O_ and *r*_H–H_ are 0.978 Å and 1.545 Å, respectively) after geometry optimization. Interestingly, a H atom also approached an O to form a –OH–; however, another H was free on the B4 site after geometry optimization. After creating a 2 × 2 × 1 supercell to find its adsorption state, the calculation indicated that the free H approaches an O atom and forms a –OH–, as shown in Figure 3j. However, on T4, the value of *E*_ads_ is positive (as the reaction is endothermic), and thus its adsorption is unstable. In Termination II, the values of *r*_H–H_ and *E*_dis_ are all almost identical to those of free H_2_, and the calculated *E*_ads_ (−0.301 eV) was less than −0.415 eV on T5, which indicated that the adsorption process was physical [30]. However, physical adsorption needs to consider dispersion (van der Waals) interactions [31,32,33]; especially, when the adsorbed molecules are larger (e.g., water, methane, benzene adsorption). These are not to be neglected; however, as the focus of this article was to investigate the chemisorbed species, physisorption was not pursued further.

The calculated capture energy of the surface for a H_2_O molecule (−0.781 eV) undertaken during further analysis of the interaction forming a H_2_O molecule on the top of O with the surface showed a weak chemical adsorption. Here, the definition of the surface oxygen vacancy formation energy is as follows [34]:

(4) EVf=Evac−Eo+12(EO2+∆hO2o)

where $E_{\mathrm{vac}}$ and $E_{o}$ are the energies of the LaNiO_3_(001) surface; with and without an oxygen vacancy, respectively. $E_{\mathrm{vac}}$ is the result of considering spin polarization.$\;E_{O_{2}}$ is the calculated energy of the O_2_ molecule, and $∆h_{O_{2}}^{o}$ is a correction term that accounts for errors that do not cancel between the treatment of oxygen in the gas and solid phases. The energy correction for O_2_ molecule is −1.36 eV [35].

Therefore, the calculated surface oxygen vacancy formation energy on T3 was −1.44 eV based on the definition, and the calculation result was slightly larger than that of the capture energy of the surface for a H_2_O molecule (−0.781 eV), which indicated that it was easy to form an oxygen vacancy on T3. The calculated Mulliken analysis of LaNiO_3_(001)/H_2_ on T3 is listed in Table 3, where O1, O2 and O3 represent three O atoms of the (001) surface. H1 and H2 indicate the two H atoms in a H_2_ molecule. The result indicated that after adsorption, the number of charges in the surface were reduced and electrons transferred from the H_2_O molecule to the surface. As the calculated bond populations of the H_2_O molecule with surface atoms are small, this means that the bonds are very weak; however, the large bond population of H–O in the H_2_O molecule means that the bond is very strong so that the H_2_O molecule could be separated from the surface. This result was supported by the electron and bond populations of LaNiO_3_(001)/H_2_ on T3. In conclusion, the interaction of a H_2_O molecule with the surface was weaker, therefore it was easy to separate from the surface to form an O vacancy. Rodriguez et al. [36] believed that the interaction between H_2_ and O vacancies are complex and that O vacancies affected the chemistry of H_2_ on the surface.

### 3.2. Chemical Process of Dissociation and Adsorption for H_2_ Molecules

The prerequisite for the reaction of a H_2_ molecule with the LaNiO_3_(001) surface is that the H_2_ molecule has to dissociate into two H atoms. Subsequently, further studies on the transition states and dissociation energy barrier of two types of dissociation processes of H_2_ molecules on the LaNiO_3_(001) surface were conducted by combining linear synchronous transit and quadratic synchronous transit. The initial structure of H_2_ molecules on the LaNiO_3_(001) surface are at T3 or B3 sites, and the final structure consisted of H_2_ molecules on the LaNiO_3_(001) surface after dissociation and adsorption. The transition states of the two dissociation processes and the activation energy barrier and reaction energy were obtained, and shown in Table 4. The H–H bond length in the transition state was somewhat stretched, the energy of the resulting structure was lower than that of the reactant, and the two processes were exothermic reactions. The results show that there is a certain reaction energy barrier in the dissociation and adsorption processes in both cases, which indicates that the reaction can be difficult to perform spontaneously and needs to be conducted under certain conditions such as heating or illuminating. From the optimized structure, we find that crystalloid defects are produced at the T3 site when two H atoms adsorb the same O atom to form a H_2_O molecule and form an oxygen vacancy after escaping from the surface. At the B3 site, two H atoms are adsorbed on two O atoms individually, thus, forming a –OH group. Comparison of the two types of dissociation and diffusion processes led to the following results: First, the activation energy barrier from the reactant to transition state at the T3 site was −0.869 eV, which meant that the reaction could easily occur; second, the activation energy barrier was −1.282 eV, which was slightly higher than the former case and contradicted the conclusion that optimal adsorption occurs at the B3 site. To determine the optimal adsorption site, the adsorption energies of a H_2_ molecule and H atom of an oxygen vacancy were calculated. The results indicated that adsorption would not occur in an oxygen vacancy for a H_2_ molecule, rather it escaped from the surface as the adsorption energy was only −0.228 eV. In contrast, when the H atom was located 2.458 Å or 3.455 Å from the surface, two H atoms both attached to a Ni atom in the vacancy due to adsorption in the optimized structure where the adsorption energy is −3.183 eV. Overall, the T3 site had a lower energy barrier for dissociation and adsorption; however, it is more difficult for the adsorption of a H_2_ molecule at this site because of the formed oxygen vacancy. Consequently, the B3 site was taken as the optimal adsorption site.

### 3.3. Analysis of Charge Population

Bonding strength among atoms is quantitatively analyzed based on charge population, and the formation of a chemical bond occurs via electron density redistribution among atoms such that the entire system achieves the lowest energy state [37]. When H_2_ adsorbs on the LaNiO_3_ surface with charge transfer, the electronic structure changes. Therefore, information about the interaction of H and the surface can be obtained by analyzing the Mulliken charge before and after adsorption. The Mulliken analysis was investigated through the projection of the plane-wave solutions onto a localized basis set [38,39,40,41]. The charge population was analyzed on B3 as it was the most stable structure in Termination I following geometry optimization. The charge populations on B3 are listed in Table 5, where, s, p and d refer to orbitals. This table shows that the population and the number of negative net charges of the O 2p orbital increase; the population of the H 1s orbital decreases; and the number of net charges significantly increases. This demonstrates that the electron of the H 1s orbital transfers to the O 2p orbital and that the H–O bond is clearly a covalent bond; subsequently, change to the other orbitals is minimal. To further analyze the bonding characteristics among atoms on B3, Table 6 lists the bond populations and bond lengths of atoms. Table 6 shows that the charge population of O–Ni clearly decreases and its bond length increases after geometry optimization. Therefore, the interaction of O–Ni is weak, but the charge population of H–O remarkably improves so that the interaction of H–O is strengthened.

### 3.4. Analysis of Electron Localization Function

**Figure 4:**
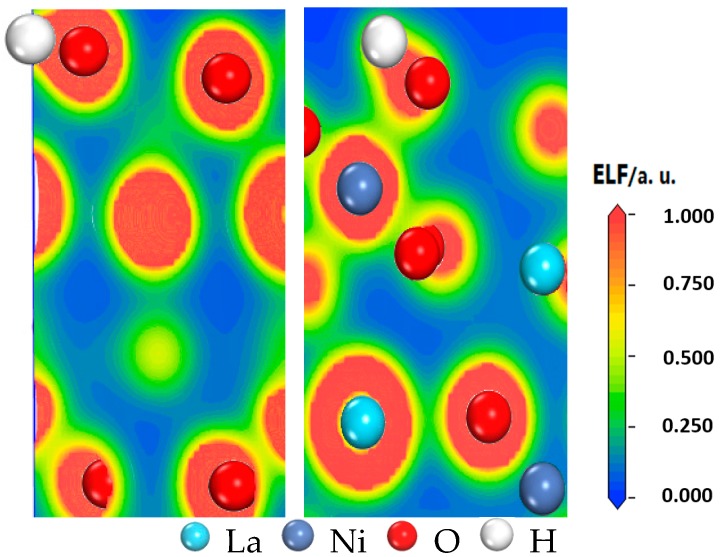
Electron localization function of LaNiO_3_(001)/H_2_ on B3 after geometry optimization of the structure.

The electron localization function (ELF) is a tool for discussing charge transfer. Becke and Edgecombe [42] proposed a method for calculating local electron distribution, which is signified by graphs. This method analyzes electrons near the nuclear area, combination bonding area and the lone pair electrons of a system, and then further analyzes the characteristics and types of chemical bonds [43]. In Figure 4, the electron density distribution of H–O on B3 is shown; here, highly localized electrons show the strongest covalent bond on ELF = 1, (red parts), a metallic bond on ELF = 0.5 and stronger ionic bonding on 0 ≤ ELF < 0.5 [43]. As shown, the electron density is intense between H and O and is clearly biased toward the O atom, which indicates that H loses an electron and O gains an electron so that their effective charges are positive and negative, respectively. Furthermore, an electron density overlap clearly exists between H and O, and H–O is in the red area. Thus, the H–O bond is a typical covalent bond, which is consistent with the previous discussion of charge population.

### 3.5. Analysis of Density of States

**Figure 5:**
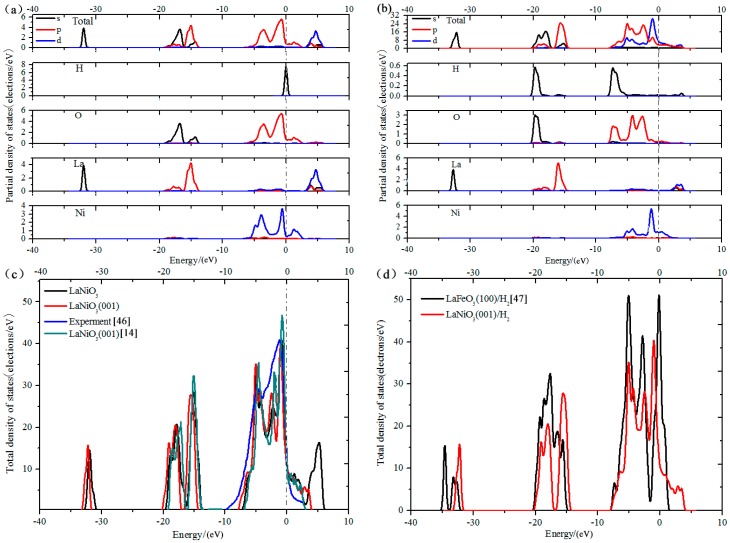
Total and partial densities of state of LaNiO_3_(001)/H_2_ on B3. (**a**) Density of states (DOS) before adsorption; (**b**) DOS after adsorption; (**c**) The theoretical calculation and experimental results [14,46]; (**d**) Comparison of total densities of state of LaNiO_3_ and LaFeO_3_ [47]. B3 represents the adsorption site of H_2_ molecule in the O–O Bridge.

Density of states (DOS) reflects the number of states for the unit energy, and is important for analyzing bonding among atoms and material properties. Therefore, the analysis of DOS can further the understanding of the interaction of H and surface atoms. The total and partial DOS of LaNiO_3_(001)/H_2_ are shown in Figure 5, where an energy of zero corresponds to the Fermi level. Figure 5a presents the DOS prior to adsorption, and it can be observed that there is no band gap near the Fermi level. Consequently, it indicates metal properties and the highest occupied state of the surface occurs in the range of −6 to 2.5 eV—mainly due to the O 2p and Ni 3d orbits—which is principally similar to the conclusion of Guan et al. [14] who stated “there is no band gap in the LaNiO_3_(001) surface and the highest occupied state of the surface is from O 2p and Ni 3d orbits”.

Lee et al. [44] and Sarma et al. [45] reached the same conclusion. Sarma et al. [45] reported electronic structure calculations of the perovskite oxides LaMO_3_ (M = Ti to Ni) using the tight-binding linear-muffin tin-orbital method. When a H_2_ molecule is inserted on the (001) surface, significant changes occurred in the total and partial states of each atom, as shown in Figure 5b. Consequently, the DOS of H was highly dispersed and the highest occupied state moved slightly toward a deep level. This illustrates that an interaction exists between H and the surface. Moreover, the energy levels of the H 1s and O 2p orbitals are broadened in the DOS, which indicates that the interaction of H and the crystal face originates from the H and O atoms of the surface. In addition, the H 1s and O 2p orbitals overlap, and the existence of an apparent resonance after adsorption shows a covalent bond between H and O. The atomic Mulliken charges and average overlap population for the H–O bond were also calculated to qualitatively analyze the mechanism of hydrogen storage, as listed in Table 4 and Table 5.

The conductivity of a material can be evaluated through its DOS. The theoretical calculation and experimental results [46] are in good agreement, and shown in Figure 5c. The results indicate that bands overlap with each other, which indicates good electrical conductivity before and after adsorption. For LaNiO_3_(001)/H_2_, the width of the conduction band decreases approximately 2.2 eV and the state density of the electron moves to a lower level. Furthermore, the DOS peaks strengthen near the Fermi level where the chance of obtaining an electron increases, and indicates that the electrical conductivity of the LaNiO_3_(001)/H_2_ system strengthens after adsorption. This result is due to the electronic contribution of H 1s and O 2p orbitals, which enhances electron orbital hybridization and rearranges the distribution of electron density. In addition, Figure 5 presents a comparison of the total DOS of LaNiO_3_ and LaFeO_3_ [47]. As shown, the conduction band of the LaNiO_3_(001)/H_2_ system is larger, and there is a flat area near the Fermi level and an obvious peak across the Fermi energy level, compared to the lack of peak across the Fermi level for the LaFeO_3_(100)/H_2_ system. The conductivity calculated by Deng et al. [1] is LaNiO_3_ > LaCoO_3_ > LaCrO_3_ > LaFeO_3_; whereas when a H_2_ molecule is inserted in the system, the calculated conductivity of LaNiO_3_(001)/H_2_ is better than that of the LaFeO_3_(100)/H_2_ system.

## 4. Conclusions

The calculated surface energy indicated that the LaNiO_3_(001) surface was the most stable surface. Subsequently, the adsorption of H_2_ on this surface was calculated and analyzed. The conclusions are summarized as follows:

Three types of adsorption were found on the surface. First, H_2_ was placed on the top of O (T1, T2, and T3), where the optimization results revealed that the H_2_ molecules were dissociated and that the H atoms tended to bond at the tops of two O atoms, thus forming two –OH at these sites. Second, H_2_ was located on the O bridge (B1, B2, B3, and B4), and results indicated that H atoms tended to bond to the same O and form one H_2_O molecule. In the above two ways, H_2_ was primarily adsorbed via chemical adsorption. Finally, there were also some physical adsorption sites, for example, the top of La (T4).

On T3, the interaction for the formation of H_2_O and the (001) surface was weaker. Thus, H_2_O was easy to separate from the surface and generate O vacancies according to the analysis of atomic and bond populations before and after adsorption on T3.

Based on the analysis of the electronic structure of LaNiO_3_(001)/H_2_ on B3, the H_2_ molecule completely dissociated and formed –OH with the O atom from the surface, and was followed by the interaction of H and the surface, which mainly originated from the contribution of H 1s and O 2p orbitals. H–O was found to be a typical covalent bond.

There was no band gap, and the contribution of the highest occupied state was from O 2p and Ni 3d orbitals. The conductivity of the LaNiO_3_(001) system was stronger after adsorption, according to the analysis of the total DOS for the (001) surface before and after adsorption. Additionally, the conductivity of the LaNiO_3_/H_2_ system was better than that of the LaFeO_3_/H_2_ system based on the comparison of their total DOS.

## Figures and Tables

**Table 1:** The calculated surface energies (eV/Å^2^) of different LaNiO_3_ surfaces.

Termination I (001)	Termination II (001)	(001) Ref. [15]	(110)	(101)	(011)	(111)	(100)	(001)
1.97	1.84	2.03	4.23	2.25	5.04	6.61	6.10	6.67

**Table 2:** The calculated geometry and energy parameters of LaNiO_3_(001)/H_2_ after geometry optimization. The experimental values are also included [27,29].

Type	Initial Position (H_2_)	*r*_H–H_ (Å)	*r*_H–O_ (Å)	*r*_H–Ni_ (Å)	*r*_H–La_ (Å)	*E*_ads_ (eV)	*E*_dis_ (eV)
Model I	T1	1.678	1.021	2.368	4.367	−1.463	0.955
	T2	1.675	0.995	2.219	3.036	−1.517	0.965
	T3	1.636	0.994	2.359	2.919	−2.074	1.105
	T4	0.757	4.153	5.310	2.951	0.045	4.525
	B1	3.104	0.982	2.257	3.210	−2.106	−1.678
	B2	2.907	0.983	2.605	2.975	−1.397	−1.520
	B3	3.047	1.003	3.035	2.901	−2.822	−1.638
	B4	2.847	0.992	2.958	3.038	−2.593	0.017
	V	3.061	0.982	2.250	3.225	−2.816	−1.648
Model II	T5	0.878	3.213	1.586	6.482	−0.301	4.313
Experiment	H_2_O [29]	1.545	0.978	-	-	-	-
	H_2_ [27]	0.752	-	-	-	-	4.48

**Table 3:** The calculated electron populations and bond populations of LaNiO_3_(001)/H_2_ on T3. T3 represents the adsorption site of H_2_ molecule in the O Top.

Atom	Electron Population (e)		Bond	Bond Population (e)	
	Before Adsorption	After Adsorption		Before Adsorption	After Adsorption
O1	−0.64	−0.72	O3–Ni	0.05	0.00
O2	−0.64	−0.72	O3–La	0.25	0.05
O3	−0.64	−0.81	O1–O3	−0.03	−0.07
La	1.47	1.34	H1–O1	-	0.10
Ni	0.56	0.40	H2–O2	-	0.03
H1	-	0.37	H2–O3	-	0.63
H2	-	0.34	H1–O3	-	0.68

**Table 4:** Energy parameters of two types of chemical adsorption.

Adsorption Site	Barrier from Reactant (eV)	Barrier from Product (eV)	Energy of Reaction (eV)
T3	−0.869	−2.833	−1.964
B3	−1.282	−3.789	−2.507

**Table 5:** The calculated electron populations of LaNiO_3_(001)/H_2_ on B3.

Atom	Before Adsorption (e)				After Adsorption (e)			
	s	p	d	Charge	s	p	d	Charge
O	1.90	4.75	-	−0.64	1.87	4.91	-	−0.78
O	1.90	4.75	-	−0.64	1.84	4.99	-	−0.83
La	2.23	6.13	1.17	1.47	2.34	6.12	1.19	1.35
Ni	0.40	0.68	8.57	0.30	0.40	0.66	8.66	0.28
H1	1.00	-	-	-	0.70	-	-	0.30
H2	1.00	-	-	-	0.67	-	-	0.33

**Table 6:** The calculated bond populations of LaNiO_3_(001)/H_2_ on B3 after adsorption.

Bond	Population (e)		Length (Å)	
	Before Adsorption	After Adsorption	Before Adsorption	After Adsorption
O1–Ni	0.40	0.22	1.832	2.168
O2–Ni	0.41	0.31	1.830	1.984
H2–O1	-	0.67	-	0.981
H1–O2	-	0.66	-	1.003
O1–La	0.24	0.25	2.247	2.504
O2–La	0.25	0.25	2.797	2.504
H2–Ni	-	–0.19	-	2.250
